# Bone Mineral Is More Heterogeneously Distributed in the Femoral Heads of Osteoporotic and Diabetic Patients: A Pilot Study

**DOI:** 10.1002/jbm4.10253

**Published:** 2019-12-11

**Authors:** Eoin Parle, Sherdya Tio, Annie Behre, John J Carey, Colin G Murphy, Timothy F O'Brien, William A Curtin, Stephen R Kearns, John P McCabe, Cynthia M Coleman, Ted J Vaughan, Laoise M McNamara

**Affiliations:** ^1^ Department of Biomedical Engineering National University of Ireland Galway Galway Ireland; ^2^ Department of Bioengineering Lehigh University Bethlehem PA USA; ^3^ Department of Rheumatology Galway University Hospitals Galway Ireland; ^4^ Department of Orthopaedics Galway University Hospitals Galway Ireland; ^5^ Department of Endocrinology Galway University Hospitals Galway Ireland

**Keywords:** DIABETES, MECHANICAL PROPERTIES, MICROARCHITECTURE, MINERAL HETEROGENEITY, OSTEOARTHRITIS, OSTEOPOROSIS

## Abstract

Osteoporosis is associated with systemic bone loss, leading to a significant deterioration of bone microarchitecture and an increased fracture risk. Although recent studies have shown that the distribution of bone mineral becomes more heterogeneous because of estrogen deficiency in animal models of osteoporosis, it is not known whether osteoporosis alters mineral distribution in human bone. Type 2 diabetes mellitus (T2DM) can also increase bone fracture risk and is associated with impaired bone cell function, compromised collagen structure, and reduced mechanical properties. However, it is not known whether alterations in mineral distribution arise in diabetic (DB) patients’ bone. In this study, we quantify mineral content distribution and tissue microarchitecture (by μCT) and mechanical properties (by compression testing) of cancellous bone from femoral heads of osteoporotic (OP; *n* = 10), DB (*n* = 7), and osteoarthritic (OA; *n* = 7) patients. We report that though OP cancellous bone has significantly deteriorated compressive mechanical properties and significantly compromised microarchitecture compared with OA controls, there is also a significant increase in the mean mineral content. Moreover, the heterogeneity of the mineral content in OP bone is significantly higher than controls (+25%) and is explained by a significant increase in bone volume at high mineral levels. We propose that these mineral alterations act to exacerbate the already reduced bone quality caused by reduced cancellous bone volume during osteoporosis. We show for the first time that cancellous bone mineralization is significantly more heterogeneous (+26%) in patients presenting with T2DM compared with OA (non‐DB) controls, and that this heterogeneity is characterized by a significant increase in bone volume at low mineral levels. Despite these mineralization changes, bone microarchitecture and mechanical properties are not significantly different between OA groups with and without T2DM. Nonetheless, the observed alterations in mineral heterogeneity may play an important tissue‐level role in bone fragility associated with OP and DB bone. © 2019 The Authors. *JBMR Plus* published by Wiley Periodicals, Inc. on behalf of American Society for Bone and Mineral Research.

## Introduction

Osteoporosis is a metabolic bone disease affecting over 200 million people worldwide, and is becoming increasingly prevalent because of an aging population.^1^ The economic burden of treatment is projected to reach $77 billion in Europe by 2050.^2^ During osteoporosis the levels of circulating estrogen in the blood are deficient, which increases the number and resorption activity of osteoclasts^3^ and decreases bone formation caused by osteoblast apoptosis,^4^, ^5^ ultimately leading to bone loss and fracture. The disease has been long regarded to be a disease of bone loss because bone mass and microarchitecture are depleted,^6^, ^7^, ^8^, ^9^, ^10^ and these are both correlated to compromised bone tissue mechanical properties and increased fracture risk.^11^, ^12^ Nonetheless, antiresorptive therapies designed to inhibit bone loss only reduce osteoporosis fractures by 50%.^1^, ^13^

Although bone loss is of primary concern, other important changes in bone tissue also arise. In particular, secondary thickening of trabeculae has been reported,^10^ and bone matrix properties and tissue mineralization are altered within the femora of rat and ovine models of osteoporosis.^14^, ^15^, ^16^, ^17^ Interestingly, although estrogen deficiency is systemic, changes in bone tissue mineralization are more prevalent at specific anatomical regions.^15^ Moreover, bone loss and compositional changes are temporally distinct, and it has been proposed that secondary changes in trabecular mineralization might be activated as a compensatory response to increased loading of bone cells imposed by the initial bone loss.^15^, ^18^, ^19^ Others have also reported increased mineralization of trabecular bone in human vertebrae^20^ and the iliac crest,^16^, ^21^ whereas conversely it has been reported that trabecular bone matrix mineralization is decreased with osteoporosis (also in the iliac crest), usually accompanied with increased mineral heterogeneity.^22^, ^23^, ^24^, ^25^ However, how osteoporosis alters mineral distribution at the femoral head, a site susceptible to osteoporosis in human bone, is not yet fully understood.

Type 2 diabetes mellitus (T2DM) is a chronic disease that is associated with high blood sugar caused by insulin resistance, which also increases the likelihood of bone fractures by up to 69%.^26^ However, unlike osteoporosis, this risk of fracture is not associated with changes that can be predicted by standard clinical BMD or FRAX (fracture prediction tool^27^, ^28^). In fact, studies have reported that patients with T2DM have normal to high BMD by DEXA.^29^, ^30^ Recent studies have found that the trabecular microarchitecture and mechanical properties of cancellous bone of T2DM (measured by cyclic reference point indentation and unconfined compression) are indistinguishable from osteoarthritic (OA) controls,^31^ whereas the apparent stiffness and yield strength were increased in cancellous cores from the femoral neck of patients with T2DM (and osteoarthritis) compared with OA controls.^32^ Impaired mechanical properties (decreased strength, stiffness, and toughness) have been reported for hyperglycemic mouse femurs tested in bending.^33^ Although fracture risk might be attributed to an increased risk of falls caused by other complications associated with T2DM (eg, obesity, poor balance, reduced muscle quality, slight problems brought on by hyperglycemia), important cellular and compositional changes also occur. In particular, patients with T2DM demonstrate impaired skeletal adaptive responses to loading,^34^ and bone formation markers are reduced in postmenopausal women with T2DM compared with those without T2DM.^35^ Rat and mouse models of T2DM have been developed^33^, ^36^, ^37^, ^38^, ^39^; using such models, it has been reported that osteoblast apoptosis and impaired osteoblast function occur and that osteoclast‐mediated bone resorption is increased as well.^38^, ^40^ T2DM in humans has also been associated with increased cortical porosity,^29^, ^41^, ^42^, ^43^ altered trabecular spacing,^32^, ^44^ and reduced cortical thickness.^29^, ^43^ At a molecular level, T2DM leads to perturbations in collagen cross‐linking,^45^ in particular, a significant reduction in beneficial enzymatic collagen cross‐links and an increase in disadvantageous non‐enzymatic collagen cross‐links,^46^ known as advanced glycation endproducts (AGEs). These may play an important role in bone fracture susceptibility in patients with T2DM and, because the collagen matrix provides the template for mineral deposition and may direct nucleation, alterations in collagen likely result in a corresponding change in tissue mineralization. However, it is not yet known whether alterations in mineral distribution arise in DB human bone, and what role such changes might play in T2DM‐related fracture risk.

In this study, our objective was to examine the cancellous bone from human femoral heads of (i) **OA** controls; (ii) OA patients with type 2 diabetes mellitus (DB); and (iii) osteoporotic (OP) patients. We quantified mineral content and mineral heterogeneity from bone mineral density distribution (BMDD) analysis; bone morphometry and microarchitecture were obtained through μCT analysis, and we correlated our findings to bone mechanical properties, specifically strength, stiffness, and loading energies.

## Patients and Methods

### Bone samples

Approval for this study was granted by the Clinical Research Ethics Committee, Galway University Hospitals, Galway, Ireland. All subjects provided written informed consent prior to participation in the study. Femoral head samples were obtained from 24 patients (aged 55 to 90 years) undergoing elective total hip replacement (THR) for chronic osteoarthritis or emergency THR or hemiarthroplasty surgery following a fragility fracture of the hip at two Galway hospital sites, Merlin Park University Hospital and University Hospital Galway. OA patients were chosen as a surrogate control group as it was not feasible to obtain sufficient young, healthy human femoral head tissue. Patients were categorized as follows: (i) OA (*n* = 7): patients undergoing THR because of chronic pain caused by osteoarthritis of the hip joint; (ii) DB (*n* = 7): OA patients undergoing THR, also clinically diagnosed with T2DM (by a consistently elevated HbA1c score (HbA1c >48, duration of T2DM: 9.8 ± 5.5 years); and (iii) OP (*n* = 10): patients undergoing THR/hemiarthroplasty caused by an acute fragility fracture of the hip. OA and DB groups did not present with fragility fractures. Details of age and gender are summarized in Table 1. None of the patients examined had any recorded comorbidities or were on medications known to affect bone metabolism (eg, glucocorticoids, antiretroviral medications, bisphosphonates, teriparatide, or denosumab).

**Table 1:** Age and Gender Details for Each Patient Group

Patient group	*N*	Cores analyzed	Age range (mean ± SD)
OA	7 (4 M, 3 F)	50	56–76 (65.1 ± 7.7)
DB	7 (4 M, 3 F)	56	58–75 (67.1 ± 7.4)
OP	10 (1 M, 9 F)	69	55–90 (71.2 ± 10.4)

Groups did not differ significantly in age (ANOVA analysis *p* = 0.43).

OA = osteoarthritis; DB = type 2 diabetes mellitus; OP = osteoporosis; M = male; F = female.

During surgery, upon removal from the patient, femoral heads were wrapped in PBS‐soaked gauze and stored in a sterile container. Samples were then frozen at −20°C before processing. Cuboid cores of dimensions approximately 13 × 5 × 5 mm were sectioned from the central region of each femoral head. Eight cores were taken from the central region of the femoral head of intact samples (OA, DB); however, because of the fragility fracture present in OP bone, depending on the condition of the bone, between four and eight cores could be obtained from each femoral head for some OP patients. The top 5 mm of subchondral bone was removed, as osteoarthritis can significantly alter its microstructure.^47^ All cores were subject to the same μCT scanning and mechanical testing protocol. Only the central region of the femoral head was analyzed in this study, as it was consistently observed to be the densest and most uniform region of the bone.

**Figure 1:**
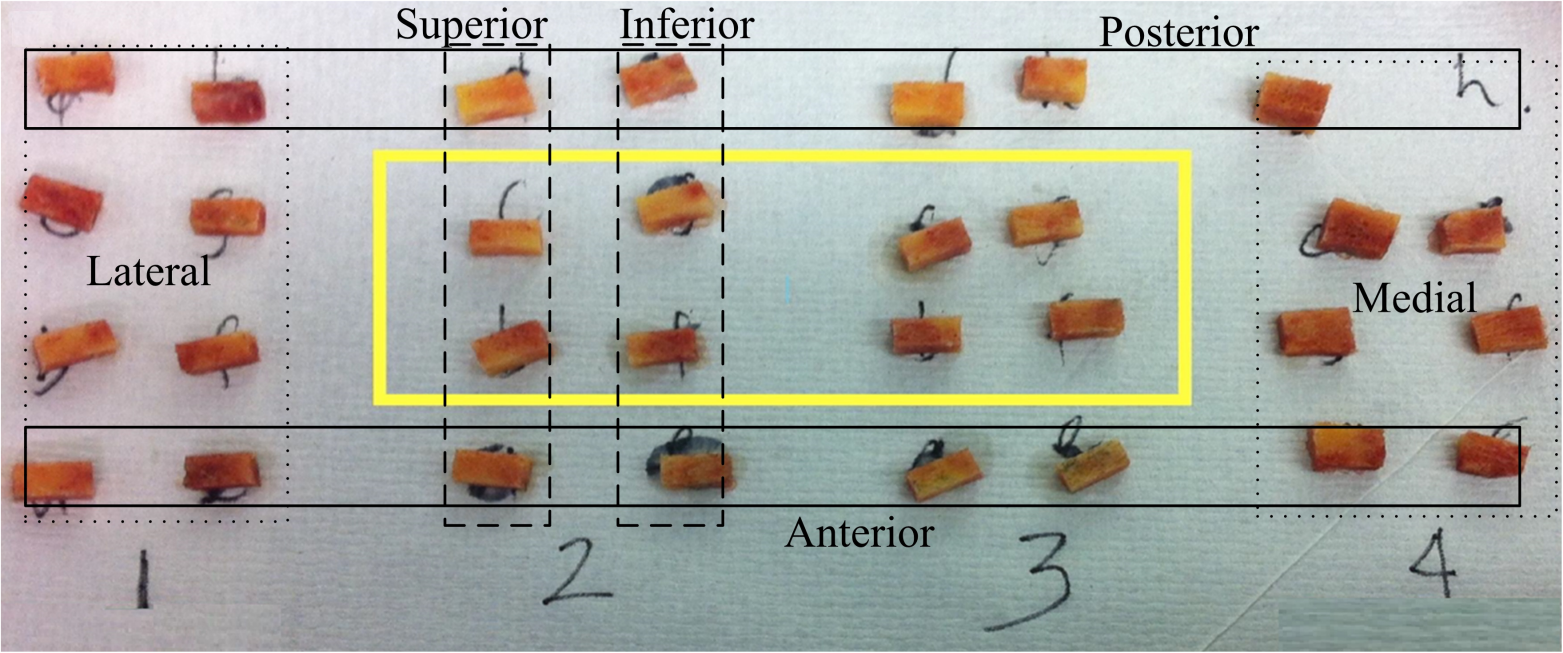
Femoral heads were cut into cuboid cores for μCT scanning and compression testing. Only cores from the central region (yellow box) were analyzed for each femoral head.

Cores were cut at an angle, such that the majority of the trabeculae were oriented parallel to the loading direction during compression testing. Preliminary analysis (using output variables “Anisotropy Parameters” H1, H2, and H3 from μCT evaluations; value >1 indicating anisotropy) was carried out to ensure our cutting technique resulted in cores with the majority of trabeculae oriented parallel to the loading direction. Once established, anisotropy was not checked for each core or analyzed as a variable for this study. Cutting was performed under irrigation using a Buehler Isomet Low‐Speed Saw (Buehler, Lake Bluff, IL, USA) fitted with a 5″ diamond wafering blade at speeds of approximately 40 rpm. This allowed accurate linear cutting of the bone into cuboid shapes required for compression testing (see Fig. 1), and the low cutting speed and irrigation minimized the possibility of thermal damage.

### CT scanning

Immediately after cutting, each core was scanned by μCT (Scanco μCT100; Scanco Medical AG, Brüttisellen, Switzerland) at 17.2‐μm voxel size with energy intensity at 70 kVp, 114 μA, 8 W; using a 0.1‐mm aluminum filter to minimize beam hardening; and an integration time of 500 ms; while submerged in PBS to prevent dehydration. Projected images were reconstructed and compiled using Scanco vendor software. Weekly calibration of the μCT machine using hydroxyapatite (HA) phantoms ensured consistency between scans. Calibration involved quality control (QC) scanning of phantom rods of 0, 100, 200, 400, and 800 mg HA/cm^3^ (milligrams hydroxyapatite per cubic centimeter) density, and ensuring the results given from the QC scan evaluations matched these densities. This ensured the accuracy of mineralization results from scan evaluations.

Volumes of interest (VOIs) were contoured manually from each bone core scan at a distance of >1 mm from the bone surface to avoid bone particles and dust that remained in pores after the cutting process. VOIs were thresholded using a single global threshold of 355 mg HA/cm^3^ for all cores. The contoured gray‐scale image was “segmented” using vendor software to remove marrow (voxels with gray scale corresponding to <355 mg HA/cm^3^) and peel two pixels from the trabecular surface to avoid the partial volume effect and to isolate bone tissue. Evaluation scripts were run on segmented VOIs to quantify trabecular bone volume fraction (BV/TV), trabecular thickness (Tb.Th; mm), trabecular number (Tb.N; mm^−1^), and trabecular spacing (Tb.Sp; mm). BMDD for each bone core was evaluated from histograms of the frequency distribution of the mineral density (mg HA/cm^3^) on the segmented gray‐scale image stack of each VOI. BMDD histograms were analyzed to estimate the mode (most frequent) mineral content and the mean (average) mineral content. The mineral heterogeneity was determined from the full‐width at half‐maximum (FWHM), which is the difference between the two points on the histogram equal to exactly half of the peak value of the distribution curve. We also took measurements from the BMDD curve to determine the percentage of the bone volume (%BV) at lower levels (<700 mg HA/cm^3^) and higher levels (>1000 mg HA/cm^3^) to further quantify how the different disease states alter mineral distribution between groups. The proportion of the bone at lower (<700 mg HA/cm^3^) and higher (>1000 mg HA/cm^3^) mineral levels was calculated from the BMDD curves. These levels were chosen instead of examining levels at <5% and >95% of the mean (as in ref 48) as the basis for comparison (mean mineral content) differed significantly between groups.

### Mechanical testing

Bones were kept hydrated in PBS for 24 hours prior to testing (placed in PBS after cutting and during scanning; only taken out of PBS immediately before testing) to prevent desiccation. Samples were tested using a single column tensile/compression testing machine (Zwick/Roell, Ulm, Germany) with a 1‐kN load cell (see Fig. 1 *C*). Cores were tested in uniaxial unconfined compression between parallel aluminum plates at a rate of 2 mm/min until failure was observed. Force and displacement data recorded during the test were used together with sample dimensions to calculate apparent stress (σ, MPa) and strain (ε) for each sample. The apparent elastic modulus (Young's modulus, MPa) of cancellous cores was calculated from the slope of the linear portion of the stress–strain curve. Apparent failure strength (MPa) was deemed to be the maximum stress endured during the test. The area under the loading curve from 0.01 strain up to the strain at maximum stress endured was used to estimate the “energy to failure.” As samples failed by compressive crushing, no definitive fracture point could be identified, thus energy to fracture (a measure of toughness) could not be estimated. A measure of “post‐yield energy” was calculated from the area under a portion of the post‐yield curve (normalized by the strain experienced). This portion corresponded to the area under the loading curve for 0.004 to 0.01 strain directly after the maximum stress experienced during a test.

### Statistical analysis

A statistical power analysis was conducted using SDs observed in our tests to ensure >80% statistical power for variables analyzed. We compared the mechanical (apparent strength, apparent stiffness, energy to failure, post‐yield energy), structural (BV/TV, Tb.Th, Tb.N, Tb.Sp), and mineral properties [mean mineral content, mode (most frequent) mineral content, mineral heterogeneity as measured by FWHM, and proportion of bone volume at higher and lower mineral levels] of cancellous femoral head bones between the three different groups (OA, DB, OP). Results from all bone cores (4 to 8 depending on the condition of the bone) from each patient were averaged. One‐way ANOVA was used to determine statistical significance (*p* < 0.05) between all three groups. If significance was indicated by ANOVA, post hoc multiple comparison Student's *t* tests (for unequal sample size and unequal variance) were used to determine statistical significance (*p* < 0.05) between individual groups.

## Results

### Bone tissue microarchitecture and mechanical properties are significantly depleted in the femoral head of human OP bone compared with OA controls

**Figure 2:**
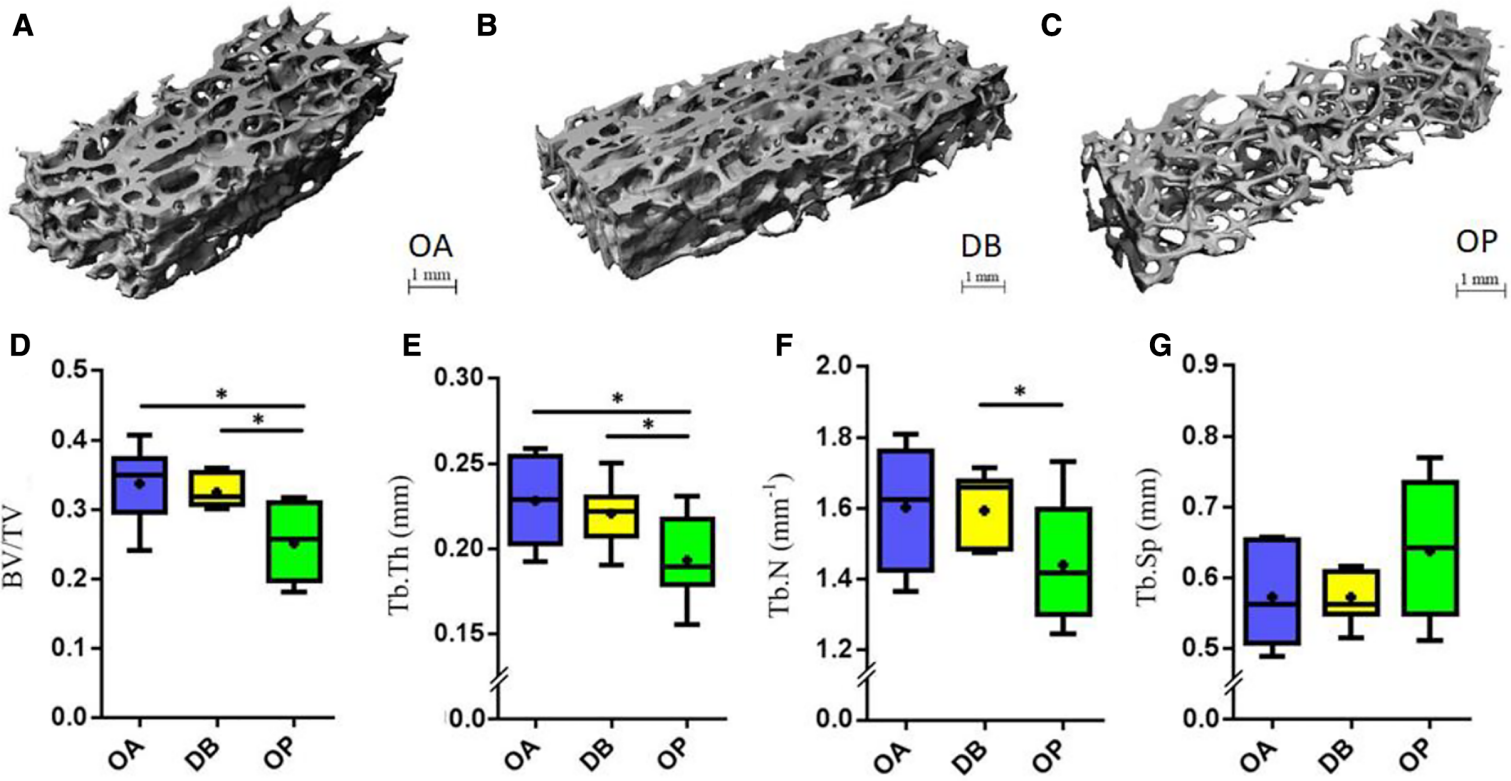
Microarchitecture. 3D‐volume renderings from μCT scans of (*A*) OA, (*B*) DB, and (*C*) OP bone. Box and whisker plots showing (*D*) bone volume fraction (BV/TV), (*E*) trabecular thickness (Tb.Th, mm), (*F*) trabecular number (Tb.N, mm^‐1^), (*G*) trabecular spacing (Tb.Sp, mm) for each patient group: OA (*n* = 7), DB (*n* = 7), and OP (*n* = 10). * Indicates significance (*p* < 0.05). OA = osteoarthritis; DB = type 2 diabetes mellitus; OP = osteoporosis.

**Figure 3:**
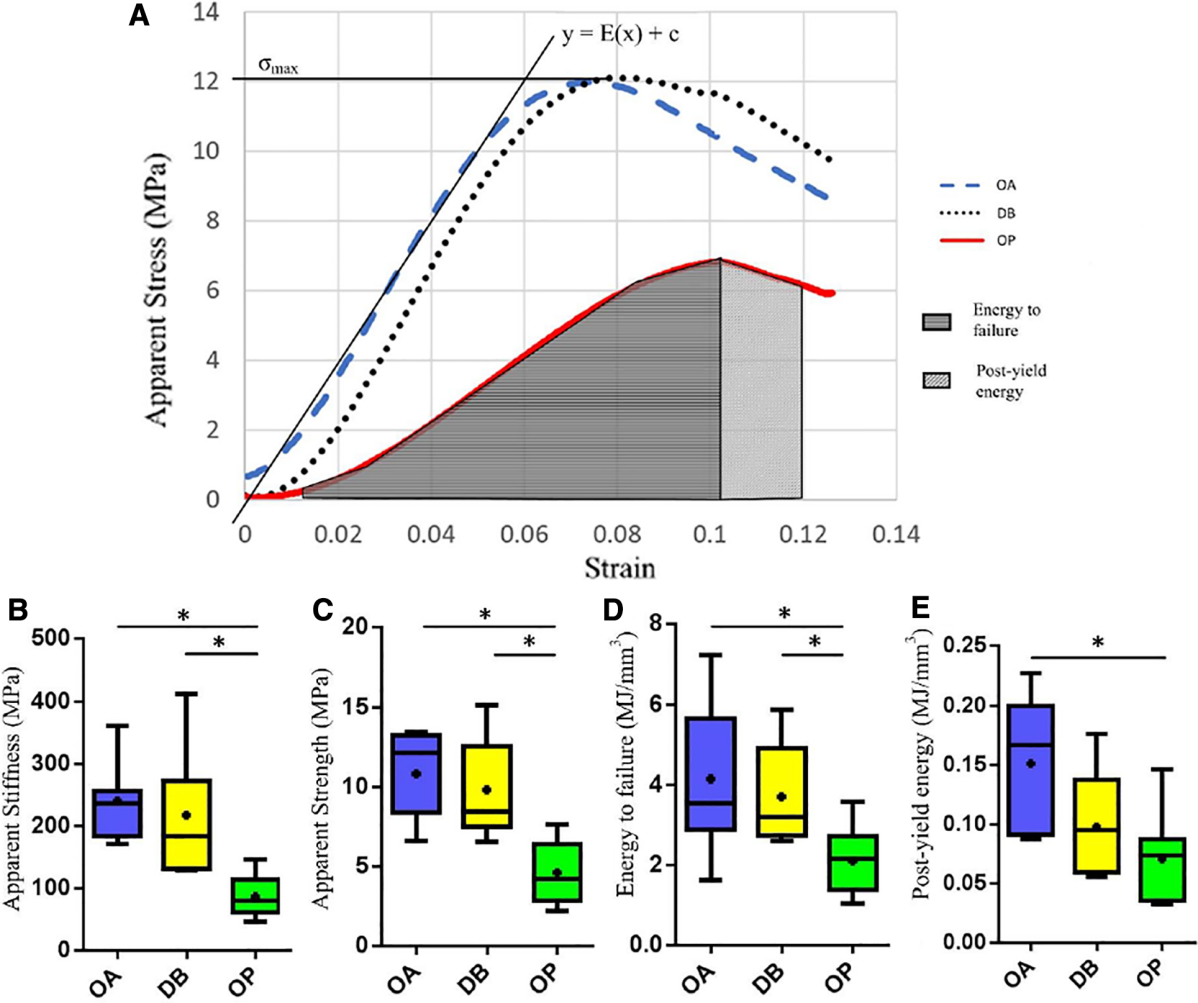
Mechanical data. (*A*) Loading curves representative of each patient group. Apparent strength (σmax) = maximum stress experienced during testing. Slope of the loading curves were used to estimate apparent stiffness. Box and whisker plots showing (*B*) apparent stiffness, (*C*) apparent strength, (*D*) energy to failure, and (*E*) post‐yield energy for each patient group. Energy to failure was estimated from the area under the loading curve from 0.01 strain to strain at maximum stress. Post‐yield energy was estimated by calculating the area under the loading curve after maximum stress (areas under the OP curve highlighted above). For consistency, this figure was then normalized by the strain experienced (in the above case [0.06–0.05 = 0.01 strain]). OA (*n* = 7), DB (*n* = 7), and OP (*n* = 10). * Indicates significance (*p* < 0.05). OA = osteoarthritis; DB = type 2 diabetes mellitus; OP = osteoporosis.

As expected, OP patients had a significantly lower bone volume fraction compared with OA controls, which can be seen visually in the 3D‐volume renderings in Fig. 2 *A–C*. This was characterized by a significant reduction in BV/TV (−26% reduction from 0.34 to 0.25, *p* = 0.005), and Tb.Th (−15% reduction from 0.23 to 0.19 mm, *p* = 0.02), Tb.N (−10% decrease from 1.6/mm to 1.4/mm, *p* = 0.09), and Tb.Sp (+11% increase from 0.57 to 0.64 mm, *p* = 0.15) were shown to decrease and increase, respectively, for OP bone compared with OA bone, but these differences were not statistically significant (Fig. 2 *D–G*). Moreover, OP bone had a significantly lower apparent stiffness (−64% reduction from 239 to 86 MPa, *p* = 0.0003) and apparent strength (−58% reduction from 10.8 to 4.6 MPa, *p* = 0.0003) than OA controls (Fig. 3 *B,C*). Likewise, energy to failure (−50%, *p* = 0.03) and post‐yield energy (−53%, *p* = 0.007) were significantly reduced for OP bone compared with OA controls (Fig. 3 *D,E*). This represents a significantly compromised microarchitecture for OP bone compared with OA, which can be correlated to the observed significantly decreased mechanical properties.

### Bone tissue mineral distribution is more heterogeneous in the femoral head of human OP bone compared with OA controls

**Figure 4:**
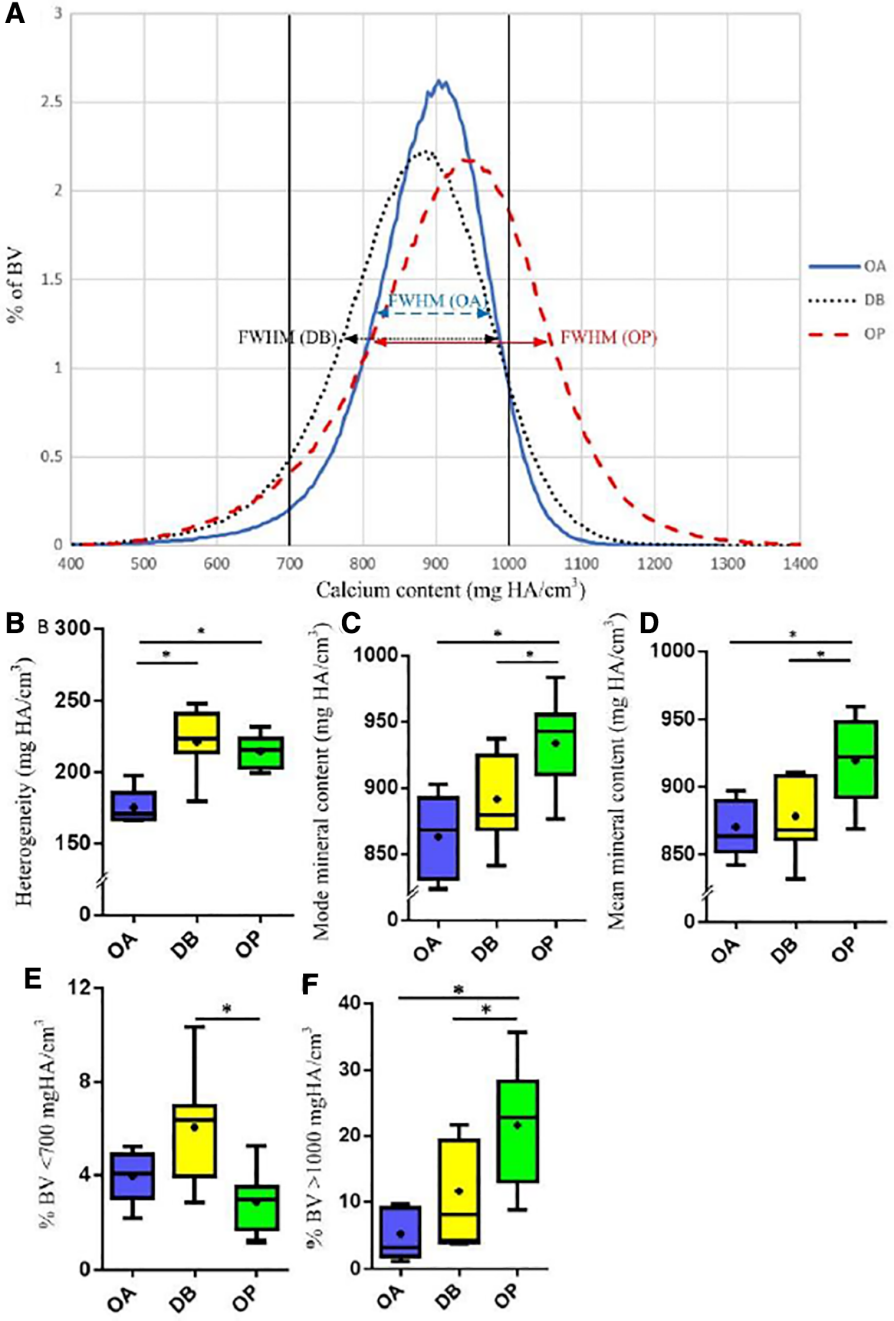
Mineral data. (*A*) Representative bone mineral density distribution (BMDD) for OA, DB, and OP bone. A histogram of % bone volume (BV) is plotted as a function of mineral content (mg HA/cm^3^) derived from gray‐scale values in the CT image. Histogram bin width = 9.5 mg HA/cm^3^. Full‐width at half‐maximum (FWHM) indicates mineral heterogeneity. FWHM is smallest for OA patients, indicating relatively homogeneous mineral content, compared with DB and OP patients. The peak (“mode”/most frequent value) for OP is shifted to the right, indicating a more highly mineralized tissue. Box and whisker plots show (*B*) mineral heterogeneity as indicated by FWHM, (*C*) mode mineral content, and (*D*) mean mineral content. Vertical lines in (*A*) define “lower” (<700 mg HA/cm^3^) and “higher” (>1000 mg HA/cm^3^) thresholds that were analyzed for each bone core. Percentages of bone volume at lower and higher levels are plotted in (*E*) and (*F*), respectively. In the graphs shown, DB shows significantly increased levels of lower mineralization compared with OP (*E*), while OP has significantly increased levels of higher mineralization compared with DB and OA (*F*). OA (*n* = 7), DB (*n* = 7), and OP (*n* = 10). * Indicates significance (*p* < 0.05). OA = osteoarthritis; DB = type 2 diabetes mellitus; OP = osteoporosis.

Our BMDD analysis revealed that the OP group had a significantly higher FWHM (+25% increase from 175 to 218 mg HA/cm^3^, *p* = 0.0002), mode (+8% increase from 863 to 933 mg HA/cm^3^_,_ *p* = 0.003), and mean mineralization (+6% increase from 870 to 919 mg HA/cm^3^, *p* = 0.001) than the OA control group (Fig. 4 *A–D*). This represents a significant increase in mineral heterogeneity, and on average, a significantly more highly mineralized cancellous bone tissue for OP patients, when compared with the OA group. OP and OA bone were not significantly different in terms of the proportion of bone at the lower mineral levels (<700 mg HA/cm^3^) examined. However, there was a significant (*p* = 0.0002) increase in the proportion of OP bone volume (21% BV) at higher mineral levels (>1000 mg HA/cm^3^) compared with OA bone volume (5% BV) (Fig. 4 *E,F*).

### Bone tissue microarchitecture and mechanical properties are unchanged in DB human bone compared with OA controls

No significant differences were detected between the DB and OA control groups in terms of bone morphometry (BV/TV, Tb.N, Tb.Th, Tb.Sp; see Fig. 2 *D–G*) or in terms of mechanical properties (strength, stiffness, or loading energies; Fig. 3 *B–E*).

### Bone tissue mineral distribution is more heterogeneous in DB human bone than in OA controls

The DB group had a significantly increased mineral heterogeneity compared with the OA group (+26% increase from 175 to 221 mg HA/cm^3^, *p* = 0.001), as indicated by the FWHM in the BMDD analysis (Fig. 4 *A,B*). No significant differences were detected between the OA and DB groups in terms of mean and mode mineral content (Fig. 4 *C,D*). On average, the DB group had more bone volume (6.1% BV) under 700 mg HA/cm^3^ than the OA group (4% BV), but this difference was not statistically significant (*p* = 0.08; see Fig. 4 *E*). The DB group also had a greater proportion of bone volume (11% BV) at higher mineral levels (>1000 mg HA/cm^3^) than the OA group (5% BV), but this difference was not statistically significant (*p* = 0.07; see Fig. 4 *F*).

### DB and OP bone differ in terms of mechanical properties and mineral content

OP bone had significantly reduced BV/TV (−23% lower, *p* = 0.002), Tb.Th (12.5% lower, *p* = 0.02), and Tb.N (9.7% lower, *p* = 0.03), matched by significantly decreased stiffness (60% lower, *p* = 0.02), strength (53% lower, *p* = 0.003), and energy to failure (43% lower, *p* = 0.049) compared with DB bone. DB and OP bone were distinctly different in terms of mineral content. OP bone showed a significantly higher mean (+5%, *p* = 0.001) and mode (+5%, *p* = 0.025) mineral content than DB bone. No significant differences were present between DB and OP bone in terms of mineral heterogeneity (FWHM, *p* = 0.8), but DB bone had a significantly higher proportion of its bone volume at lower mineral levels than OP bone (6.1% versus 2.9% BV, *p* = 0.01; Fig. 4 *E*), whereas OP bone had significantly more of its bone volume at higher levels (22% versus 12% BV, *p* = 0.02).

## Discussion

In this study, we found significant alterations in the mineral content of cancellous bone in the femoral heads of both OP and DB patients compared with OA controls. BMDD analysis revealed for the first time that the mineralization of human OP femoral bone was significantly more heterogeneous than that of OA controls, and this increase in heterogeneity was largely because of an increase in the proportion of bone at higher mineral levels. DB patients were shown to have a significant increase in mineral heterogeneity compared with OA patients, which was largely caused by an increase in the proportion of the bone at lower mineral levels. However, unlike the OP group these changes were not coupled to microarchitectural degradation or a reduction in mechanical properties.

There are some limitations to this study that must be noted. First, we only examined bone cores from the central region of the femoral head because this is the most highly loaded and strongest cancellous bone^49^ associated with the transfer of stress from the acetabulum to the femoral diaphysis.^50^ It is therefore the area of greatest density and TB.Th, and has the greatest degree of trabeculae aligned in a single direction.^51^ This region may have adapted in the primary loading direction (our testing direction)^51^ to maintain a dense core at the expense of peripheral cancellous regions, which may represent a site of fracture initiation under an impact load such as a fall. Nonetheless, it is interesting that the mineral distribution is becoming more heterogeneous within this localized region; further studies are required to delineate whether changes also arise in the peripheral regions. Second, by our methods we did not observe altered mechanical properties at the tissue level despite previous observations of increased fracture risk in DB patients at the population level, which is in keeping with the findings of a study that compared the mechanical properties (compression and Reference Point Indentation)^31^ of DB femoral cancellous bone to non‐DB OA patients, although another study reported that DB bone had increased stiffness, yield, and ultimate strength.^32^ These studies highlight the challenge of delineating the underlying mechanisms for increased fracture risk in DB patients, which might be associated with properties not distinguishable through our approach, in particular, fracture toughness. Moreover, similar to other studies,^31^, ^32^ we used OA bone as a surrogate control as no healthy human bone was available and, although we could distinguish between the two groups in terms of mineralization, this might explain the lack of difference in mechanical properties. Nano‐indentation and fracture toughness testing is thus required to further investigate whether the increased mineral heterogeneity plays a role in the propensity for DB human bone to fracture. BMI is a complicating factor in T2DM that may affect bone density but unfortunately, no BMI data were available for the patients analysed. Our peeling operation (prior to mineral content analysis) ensured our data were not skewed by the partial volume effect, which we showed to increase heterogeneity by 20% to 30% for unsegmented and unpeeled images. Although changes in mineralization at trabecular surfaces could not be captured, the approach captures important changes arising deep within the trabecula, and in fact circumvents local and temporal effects as a result of active remodeling surfaces. μCT for BMDD analysis has been used previously to analyze mineral content and distribution in rat mandibles,^48^ but the resolution is inferior to quantitative backscattered electron imaging (qBEI) and synchrotron radiation‐based μCT.^15^, ^16^ One study reported that heterogeneity measured using qBEI was not well‐correlated with μCT.^52^ However, the method of relating voxel gray levels to mineral content is the same and our resolution (17.2 μm) falls within recommendations defined for such analysis.^53^ Moreover, our approach enabled BMDD analysis of the large 3D‐VOIs in the central region of the femoral head for a large cohort of patients, rather than being limited to a single 2D‐plane as is the case for qBEI. Analysis of BMDD curves also allowed us to inspect the full spectrum of mineralization by comparing mean, peak, and heterogeneity of mineral content, whereas a focussed average tissue mineral density can mask this important information. Moreover, we show that even at these relatively low resolutions, we can detect significant differences in mineral heterogeneity between our groups; we would thus expect these differences to be even more pronounced under higher resolutions, such as those achievable using qBEI.

Previous studies have shown increased mineral heterogeneity in osteoporosis compared with healthy controls in individual trabeculae for human iliac crest and T12 vertebrae,^16^ as well as in a sheep femur^15^, ^18^ but altered bone mineral distribution has not been reported before in the human femoral head. Similar to previous studies on OP human^16^, ^20^, ^21^ and animal cancellous bone,^17^ our BMDD analysis on OP bone revealed a shift toward a more highly mineralized tissue, when compared with OA and DB groups. In OP bone, we found a pronounced increase in the proportion of the bone at higher mineral levels. It has been proposed that during osteoporosis specific trabeculae become thinner as a result of resorption whereas others become thicker,^10^ which might explain the increases in heterogeneity we report here. The lower mineral levels present may be caused by the formation of new bone matrix^54^ or thickening trabeculae. The higher mineralized tissue might be explained by osteoclast removal of the less mineralized surface, leaving a more highly mineralized core. Hyper‐mineralization caused by the infilling of osteocyte lacunae has been shown to be increased in osteoporosis^55^ and could also contribute to the observed increase in mineralization. Alternatively, this may be related to the altered mechanical environment arising after the initial bone loss. It has been proposed that bone loss would expose osteocytes within remaining trabeculae to altered stress levels, and the observed mineral alterations may occur as a direct response^9^, ^56^ in an effort to reinforce the trabeculae, but ultimately render the tissue more brittle.

We have shown for the first time a significant increase in mineral heterogeneity in DB bone compared with OA controls. This increase in mineral heterogeneity was manifested by a significant increase in the lower mineral levels examined compared with OA controls. Like OP bone, DB bone tended to have an increased proportion of its bone volume at higher mineral levels (1000 mg HA/cm^3^), albeit this increase was not statistically significant (*p* = 0.07). Similarly, our BMDD analysis showed that the mean level of mineralization in the central region of the femoral head is not altered during T2DM. To the authors' knowledge, the only other published study on BMDD in DB patients examined the femoral neck,^57^ and contrary to our findings showed that DB bone has a less heterogeneous and more highly mineralized cancellous tissue. This highlights the possibility that alterations in mineral content in DB bone may be site‐specific.

An increase in non‐enzymatic (AGEs) collagen cross‐links is known to arise in T2DM.^46^ It is possible that this could be the underlying mechanism responsible for the observed increase in mineral heterogeneity. Although the mean mineral content (correlated with strength and stiffness^58^, ^59^) remains unchanged, the increase in impaired enzymatic collagen cross‐linking and an excessive accumulation of AGEs cross‐linking observed in DB bone^45^ can impair the physiological mineralization process.^60^ The secondary mineralization process could be perturbed by alterations in the nucleation of calcium crystals in periodic gaps present within collagen fibrils, and on their surface^60^ caused by AGEs cross‐link‐related irregularities in collagen structure.^61^ Normal crystal growth within collagen fibers may also be inhibited by excessive cross‐linking. Rat models of T2DM^62^ have revealed decreased hydroxyapatite crystal perfection and decreased “mineral quality” (determined by calcium/phosphate ratio), and alterations from normal crystal size and composition have been shown to reduce the mechanical integrity of the bone.^63^

T2DM has been correlated with changes in bone structure. Cortical bone has been shown to have an increased porosity^43^ whereas cancellous bone architecture can be improved (increase in trabecular thickness^41^) or unchanged^29^ when compared with controls, but this is likely highly dependent on the area examined and the technique used. Our μCT analysis detected no significant differences at the micro‐level between DB and OA bone in terms of microarchitecture, suggesting that increased mineral heterogeneity can occur without a reduction in bone volume fraction (seen in OP). Moreover, unlike the OP group, changes in mineral heterogeneity in the DB group were not accompanied by significant differences in compressive mechanical properties. The similarities between osteoarthritis and T2DM in terms of mean mineral content and bone microarchitecture aligns with previous observations that BMD is unchanged in T2DM.^29^, ^64^

An increase in non‐enzymatic (AGEs) collagen cross‐linking may not only affect the mineralization process, but it can also reduce matrix ductility by inhibiting normal collagen fibril sliding and debonding at the calcium–collagen interface. This can stiffen the matrix, making it more brittle and increasing the load transferred to the crystal phase.^60^, ^65^, ^66^ These alterations to collagen and crystal structure may illicit a reduction in toughness that would not affect the compressive mechanical properties we examined here (strength, stiffness, energy to failure). Indeed, perturbations in collagen cross‐linking induced in vitro led to increased formation and accumulation of microdamage, resulting in dramatically reduced bone toughness.^29^, ^67^ These changes can occur independent of determinants of bone strength and stiffness such as mineral phase and microarchitecture, and without any change to BMD or gross collagen content.^46^

Although our analysis did not reveal any differences in mechanical properties between OA and DB groups, the increase in mineral heterogeneity reported here in DB patients highlights a departure from normal bone composition. A comprehensive analysis of the extracellular matrix, examining internal trabecular structure in terms of mineral discontinuities, calcium crystallinity, particle size and shape, collagen cross‐linking composition (enzymatic and non‐enzymatic) and mineral/matrix ratio may highlight the mechanisms behind the increase in mineral heterogeneity and the structural abnormalities responsible for bone fragility seen in diabetes. Coupling this with a fracture toughness analysis would reveal the extent of the embrittlement caused by increased mineral heterogeneity and improper cross‐linking and may tease out the differences between OA and DB bone, providing an enhanced understanding of the propensity for DB human bone to fracture more easily.

## Conclusion

We have shown for the first time a significant increase in mineral heterogeneity, together with a significant increase in the proportion of cancellous bone at higher mineral levels in the femoral head of OP patients compared with controls. These changes might be a secondary mechano‐biological response to bone loss, or a response to increased loading, but ultimately act to exacerbate the reduction in mechanical strength brought about by the compromised trabecular architecture. This study provides the first BMDD data on femoral heads from patients with T2DM. The observed significant increase in mineral heterogeneity is the result of a significant increase in the proportion of the bone at lower mineralization levels compared with controls. This may compromise the DB bone matrix structure, rendering it more brittle and prone to micro‐damage initiation and accumulation. With an increasing aged population, an understanding of the mechanisms underlying fracture risk is crucial. This research provides an advanced understanding of changes in bone quality in T2DM and osteoporosis, which can inform future diagnosis and treatment.

## Disclosures

The authors declare no competing interests
